# Interactions between the *κ* opioid system, corticotropin-releasing hormone and oxytocin in partner loss

**DOI:** 10.1098/rstb.2021.0061

**Published:** 2022-08-29

**Authors:** Karen L. Bales, Forrest D. Rogers

**Affiliations:** ^1^ Department of Psychology, University of California, Davis, CA 95616, USA; ^2^ Department of Neurobiology, Physiology, and Behavior, University of California, Davis, CA 95616, USA; ^3^ California National Primate Research Center, Davis, CA 95616, USA; ^4^ Princeton Neuroscience Institute, Princeton University, NJ 08540, USA; ^5^ Department of Molecular Biology, Princeton University, NJ 08540, USA

**Keywords:** oxytocin, opioids, kappa opioid receptor, partner loss, grief, separation

## Abstract

Selective adult social attachments, or ‘pair bonds’, represent central relationships for individuals in a number of social species, including humans. Loss of a pair mate has emotional consequences that may or may not diminish over time, and that often translate into impaired psychological and physical health. In this paper, we review the literature on the neuroendocrine mechanisms for the emotional consequences of partner loss, with a special focus on hypothesized interactions between oxytocin, corticotropin-releasing hormone and the *κ* opioid system.

This article is part of the theme issue ‘Interplays between oxytocin and other neuromodulators in shaping complex social behaviours’.

## 1. Introduction

Relationships define much of our lives—we see ourselves as a partner, a parent, a sibling, a friend. In particular, attachment relationships are important in helping us to regulate our emotions [1], and they have a strong and biologically significant effect on our health [2]. While originally described between infants and their mothers [3–5], the general principles of attachment relationships also apply to adult pair bonds [6–9]. These are relationships in which we have a strong, selective preference for a particular partner; in which we experience distress upon separation from that partner; and in which the partner is able to help us internally regulate our stress (‘buffering’ us from outside stressors). A large body of literature has found that neuroendocrine systems including oxytocin (OT), arginine vasopressin (AVP), dopamine (DA), corticotropin-releasing hormone (CRH) and opioids underlie the formation and maintenance of these adult attachment relationships or ‘pair bonds’ [10]. In an adjacent social context, the opioid system has also long been studied for its role in infant–mother attachment [11–14]. In this paper, we will review current knowledge about the neuroendocrine basis for distress at the loss of an adult partner. We distinguish grief from loneliness, or ‘feelings of distress and dysphoria resulting from a discrepancy between a person's desired and achieved levels of social relations' [15], in its dependence on the loss of a specific individual. We will also review interactions between the *κ* opioid receptor (KOR) system and the OT system, and how we hypothesize that these interactions could underlie the response to being separated from an attachment partner. Specifically, we hypothesize that during grief, the KOR mediates the inhibitory effects of CRH on the OT system.

## 2. Grief and partner loss in humans

The loss of an attachment partner can be devastating to an adult's psychological and physical health. Partner loss, separation, divorce [16], and the resultant experiences of grief, social isolation and loneliness have been independently associated with increased risk for stroke, heart disease and overall mortality [17–19]. For instance, in a large sample from a Finnish population, deaths from ischemic heart disease and cerebrovascular disease approximately doubled for men in the first month following the loss of their partner, with a 400% increase in those under age 65 [20]. Following the loss of a partner, bereavement has been associated with a 15% to over 200% increased rate of mortality (depending on age group) in the surviving spouse, with risk due to divorce and separation nearly as high [21]; evidence of these outcomes has been supported in many recent meta-analyses [22].

Prolonged or ‘complicated’ grief may be experienced by some after partner loss. Higher levels of suicidal ideation were correlated with levels of complicated grief in elderly bereaved individuals [23]. Prolonged grief disorder, or PGD, has been recognized for some time [24] and is already included in the ICD-11 [25]; however, PGD was only recently approved for addition to the revised DSM-V [26], due out in 2022. Draft criteria, published by the American Psychiatric Association for comment, require the symptoms to be a response to the death of a close person at least 12 months prior, followed by preoccupation with or intense longing for the person, to a significant degree nearly every day for the past month. These events are associated with other symptoms such as identity disruption or intense loneliness, to a degree of significant distress outside of social norms [27,28]. Common comorbidities include anxiety disorders, substance abuse disorders, major depressive disorder and post-traumatic stress disorder [27].

In humans, the cingulate cortex [29] and nucleus accumbens (NAcc) [30], as well as the amygdala [31] and insula [32,33], have been implicated in bereavement in functional magnetic resonance imaging studies [34]. The vast majority of studies on the endocrine correlates of prolonged grief in humans have focused on cortisol and the HPA axis, with substantial evidence that grief is associated with higher cortisol levels [35]. In humans, relationship distress is often associated with elevated plasma OT [36], particularly in women [37,38], and specifically in prolonged grief [36]. There is significant controversy over the functional meaning of plasma levels of OT, and to what extent these may be correlated with central levels, although one plausible viewpoint is that peripheral and central levels are sometimes coordinated by stressors or social stimuli [39,40]. It is possible that this elevated plasma OT, especially if it is accurately reflective of central nervous system OT, reflects a homeostatic mechanism by which humans are primed to then seek out substitutive social interactions [41,42].

## 3. Neuroendocrine systems studied in animal models

The primary neurohormones that have been studied in animal models in regard to partner loss are CRH and OT ([43]; see following sections and table 1). CRH has been framed as the primary neurohormone released in partner loss, in turn suppressing OT [52]. However, our thesis here, based on its known dysphoric effects, its relationships to CRH and OT, and the neuroendocrine changes induced by pair bonding in prairie voles, is that the *κ* opioid system is also likely to be involved, particularly in the dysphoric aspects of partner loss. The KOR is a G-coupled receptor [53] with only one known ligand, dynorphin, which also has bioactive fragments [54]. As it is activated by CRH, and in turn modulates OT, we suggest that KOR is a ‘missing link’ in our understanding of the separation response.

**Table 1.** Behavioural, hormonal and neurobiological findings from partner-separation studies of pair-bonded prairie voles and titi monkeys. Abbreviations: ACTH, adrenocorticotropic hormone; AVP, arginine vasopressin; BNST, bed nucleus of the stria terminalis; CeA, central amygdala; Cere, cerebellum; CRH, corticotropin-releasing hormone; CSF, cerebrospinal fluid; EPM, elevated plus-maze; FST, forced swim test; LS, lateral septum; MeA, medial amygdala; mRNA, messenger ribonucleic acid; NAcc, nucleus accumbens; OT, oxytocin; PAG, periaqueductal grey of the midbrain; PVN, paraventricular nucleus of the hypothalamus; SON, supraoptic nucleus of the hypothalamus; VP, ventral pallidum.

species	manipulation	duration	results	reference
prairie vole	partner separation (males)	3–5 days	increased floating in FST increased corticosterone no difference in CRH mRNA in the BNST no difference in CRH mRNA in the PVN	[44]
	partner separation (males)	3 days	increased floating in FST reduced OT mRNA in PVN OT receptor binding in NAcc shell reduced OT agonist or CRH receptor 2 antagonist in NAcc shell reverses passive coping	[45]
	partner separation (males)	4–6 days	in males that formed partner preferences, higher anxiety-like behaviour and increased pain responses	[46]
	partner separation (males)	2 weeks	partner preference elevated corticosterone no change in plasma OT or AVP	[47]
	partner separation (males)	4 weeks	no partner preference less open arm time in EPM more time in the dark side of dark box more affiliative with strangers iincreased body weight gain elevated corticosterone elevated OT, AVP, and CRH in PVN	[47]
	partner separation (males)	5 days	increased heart rate and reduced heart rate variation higher immobility and heart rate during the FST altered responsivity of heart rate to drugs	[48]
	partner separation (both sexes)	5 days	higher immobility in tail-suspension task and FST higher plasma ACTH and corticosterone	[48]
	partner separation (both sexes)	5 days	higher immobility in tail-suspension task following 10 days of chronic mild stress no change in FST higher plasma ACTH and corticosterone in separated females but not males	[49]
	partner separation (lactating females)	males removed a few days before birth	maternal care unchanged decreased time in open arms of EPM increased floating in FST no difference in CRH mRNA in the BNST elevated CRH in PVN under basal conditions	[50]
titi monkey	partner separation (males)	48 h	reduced glucose uptake in VP, LS, PVN, PAG, Cere increased plasma cortisol and insulin increased CSF OT	[51]
	partner separation (males)	2 weeks	reduced glucose uptake in CeA reduced glucose uptake in whole brain increased CSF OT and plasma insulin	[51]
	partner reunion with female partner (males)	following app. 2-week separation	reduced glucose uptake in MeA, CeA, SON, PVN increased CSF OT, plasma OT, and plasma insulin decreased CSF AVP	[51]
	stranger encounter with stranger female (males)	following app. 2-week separation	reduced glucose uptake in VP increased CSF OT, plasma insulin decreased CSF AVP	[51]

## 4. *Κ* opioids, oxytocin, corticotropin-releasing hormone and their interactions

The *κ* opioid system interacts intimately with CRH [55], affecting an array of stress- and anxiety-related behaviours, such as conditioned place aversion [56], swim stress immobility [57], startle response [58] and social defeat [59]; reviewed in [55]. Phospho-KOR-immunoreactivity is induced by CRH and by stress in numerous mouse brain structures associated with the stress response [60]. Dynorphin, which is the endogenous ligand for KOR, is released by the activation of corticotropin-releasing hormone receptor type 2 (CRHR2) [60], while pre-treatment with KOR antagonists prevents this release of stress-induced dynorphin [61,62]. Blockade of KOR prevents CRH-induced attention deficits in a five-choice serial time reaction test, in rats [63]. The relationship between CRH and KOR activation is generally viewed as unidirectional, with CRH release leading to dynorphin/KOR activation [55]. The role of corticosterone is less clear; while dynorphin knockouts show an extended elevation of corticosterone in relation to stress [58], it is viewed as less likely that glucocorticoids are acting centrally to mediate the aversive component of stress [55].

OT is a nine-amino acid peptide, primarily made in the paraventricular and supraoptic nuclei (PVN and SON) of the hypothalamus [64]. OT has one known receptor, a G-coupled receptor that can have differing effects depending on which secondary messaging system it activates [65]. OT also has numerous interactions with hormones within the hypothalamic–pituitary–adrenal axis, including CRH and glucocorticoids [66,67]. Interactions between OT and the µ opioid system have been studied as well, both in the context of pregnancy in rats [68] and in social attention in primates [69].

OT is in turn modulated by the *κ* opioid system, in ways that particularly implicate the involvement of the *κ* opioid system in the negative aspects of separation and partner loss. As noted above, KORs have been implicated in social stressors, particularly social defeat stress in California mice (*Peromyscus californicus*) [59,70,71] and C57BL/6J mice [62]. KORs are implicated in social memory, with prodynorphin knockouts in mice exhibiting an array of changes in social but not object memory [72]. KOR mRNA expression measured in human brain tissue colocalizes considerably with that of OT receptor mRNA [69]. KORs are present on OT neurons in rat hypothalamus and pituitary [73], while the manipulation of KORs alters the release of plasma OT; i.e. KOR agonists decrease plasma OT, whereas KOR antagonists increase plasma OT in rats [74,75]. In the NAcc specifically, a KOR antagonist attenuated OT-induced antinociception in rats [76].

## 5. Animal models of partner loss

Animals that form attachments provide powerful insights into the neurobiological processes of separation and bereavement. Non-human animals can maintain a broad range of important social relationships—from friendships [77], to parent–infant bonds [78], to sibling relationships [79], to pair bonds [80]. However, each species displays a subset of these relationships in accordance with its respective evolutionary and ecological history, and relatively few mammalian species actually have the capacity to form pair bonds [81]—only some species will experience ‘loneliness’ [15]. Many common laboratory species, like rats, mice, and rhesus monkeys, do not form pair bonds [81].

Here, we will focus on two pair-bonding species that have been well studied in the wild and in the laboratory, prairie voles (*Microtus ochrogaster*) and coppery titi monkeys (*Plecturocebus cupreus*). These species share the characteristics described above for human pair bonding, including preference for a specific partner [82–85], separation distress [47,51] and stress buffering by the partner [49,86–89]. They both show behaviours that are sometimes associated with pair bonding in humans and other species, such as biparental care [90,91] and behavioural synchrony [92]. They also show ‘jealousy’ [93,94], which is an emotional reaction to a threat to the relationship by a third party [95], and is a key mechanism for pair-bond maintenance.

## 6. Mechanisms of partner loss in adults: prairie voles and titi monkeys

### (a) Prairie vole studies of partner loss

Prairie voles have provided most of the evidence for the involvement of CRHR2 and OT in the neurobiology of bereavement. In male prairie voles, short separations (3–5 days) from a partner (i.e. mate) were associated with increased corticosterone, although CRH mRNA in the bed nucleus of the stria terminalis (BNST) did not change [44]. The same manipulation resulted in a reduction of OT mRNA in the PVN and of oxytocin receptor (OTR) binding in the NAcc shell [45]. Reduction of CRH signalling by an injection of CRHR2 antagonist, or an increase in OT signalling via chronic infusion into the NAcc shell, resulted in less floating (passive stress-coping) in males during the forced swim test following separation from their partners [45]. In the same study, infusion of an OT receptor antagonist into the NAcc shell increased floating, mimicking partner loss; while reducing OTR signalling with RNAi increased floating in males even while with their partners [45]. Blockade of corticotropin-releasing hormone receptor type 1 (CRHR1), as well as CRHR2, receptors also blocked passive-coping behaviours in male prairie voles [44].

In pregnant female prairie voles whose male mates were removed a few days before parturition, CRH mRNA in the BNST did not differ between groups; however, CRH mRNA expression in the PVN was elevated [50]. As in males, blockade of both CRH receptors reduced passive-coping behaviour [50].

An interesting study divided male prairie voles into one group that formed a significant partner preference for their mate and a second group that did not, based on natural individual variation [46]. Following both groups from four to six days following separation from the mate, males that had formed significant partner preferences showed effects of separation including higher anxiety-like behaviour and increased responses to pain. By contrast, males that had not shown a significant partner preference before separation did not show changes in anxiety-like behaviour or pain responses [46].

In a study of longer-term separation, paired male prairie voles were separated from their female partners for either two or four weeks. After two weeks, males still showed a preference for their partner compared to a stranger. They also had elevated corticosterone concentrations that persisted at both two-week and four-week timepoints; however, plasma OT and AVP were not altered. Following a four-week separation, male voles that had been separated from their pair mates showed increases in OT, AVP and CRH-immunoreactive cells in the PVN [96]. At this timepoint, males also failed to show a preference for their partner when given a choice between the estranged partner and a stranger female. It is worth noting that while the increase in neuropeptide immunoreactivity could be interpreted as increased production as in our model, the authors interpret them as possible decreased production associated with lower receptor availability. They also suggest the possibility of a peripheral surge of peptide, although they did not find elevated plasma OT or AVP in that study [96].

It is also worth noting that pair bonding induces changes in the opioid systems in prairie voles, and that KORs are involved in the behavioural regulation of the pair bond [93,97]. Aversion to novel same-sex animals is viewed as mate-guarding and part of behavioural maintenance of a pair bond in prairie voles [83]. Blockade of KORs, but not mu opioid receptors (MORs), in the NAcc shell reduced mate-guarding in both sexes [97]. KOR agonists given in the NAcc shell to males immediately prior to pairing resulted in aversion to the new mate and a preference for a novel female. However, over the first two weeks of cohabitation in both sexes, pair bonding induced an increase in dynorphin mRNA in the NAcc. In males only, pair bonding was also associated with lower levels of KOR in the NAcc [93]. These changes in the *κ* opioid system, while most likely assigning a selective negative valence to same-sex strangers [93], may also provide a substrate for the later expression of separation distress—i.e. elevated KORs in the NAcc that respond to separation from the partner. Prairie voles may also demonstrate a higher sensitivity to manipulation of the *κ* opioid system than rats or mice, with higher levels of KOR agonist-stimulated [^35^S]GTP*γ*S binding in forebrain areas—which could also be theoretically related to the capacity for demonstrating separation distress [98]. For additional reading on OT and opioid interactions in the context of their relation to pair-bond formation in prairie voles, we recommend the recent review [99].

### (b) Titi monkey studies of partner loss

Titi monkeys have provided a novel primate model for the neurobiology of separation and bereavement, thus bridging rodent and human studies [51,100]. Titi monkeys of both sexes respond to separation from the pair mate with increased vocalizations, locomotion and cortisol response [101,102]. They do not show this response to separation from other family members, nor does the presence of other familiar animals reduce this response [103]. We have previously carried out a positron emission tomography (PET) imaging study of short- (48 h) and long-term (2 weeks) separations in male titi monkeys [51]. In this study, we found multiple neurobiological effects of these manipulations, starting with a widespread reduction in glucose uptake in many areas associated with social engagement and motivation. These areas included the ventral pallidum, lateral septum and PVN of the hypothalamus, as well as the periaqueductal grey, which releases opioid peptides. OT in cerebrospinal fluid (CSF) is usually thought to reflect central release of OT in other species [104] and has been shown to correlate with OT content in the posterior pituitary of cynomolgus macaques, another non-human primate [105]. For titi males in both short- and long-term separation conditions, as well as following reunion with the partner or an encounter with a stranger, CSF OT was elevated. However, only in the reunion condition was plasma OT elevated: when males were reunited with their partner, they had elevated OT both centrally and peripherally, suggesting an additional release of OT coordinated with the reunion [51]. This increase in both central and peripheral OT, which was seen only with the partner and not the stranger female, is consistent with a context- and partner-specific role for OT in the separation response as a mechanism for maintaining the pair bond [40].

Opioids have also been studied in relation to separation distress in adult titi monkeys [88,106]. Titi monkey µ opioid receptor and KOR distributions were mapped and found to be similar to the distribution in other primates, thus potentially providing a relevant model for humans [107]. During a 1 hour separation from their partner, µ opioid receptor manipulation had no effect on plasma OT [88]. Although *κ* opioid agonism had little effect on separation behaviour, it led to an increase in plasma cortisol especially at the highest dose tested (0.1 mg kg^−1^ U50,488). A *κ* opioid antagonist, GNTI, was able to suppress separation-induced locomotion [106], suggesting that the KOR system supports the experience of distress related to separation in titi monkeys. OT was not measured in this study.

Results from studies of ‘grief’ or partner loss in prairie voles and titi monkeys—experiments where pair-bonded animals were separated from their mates—are summarized in table 1. Please note that we are distinguishing these studies from other studies in which social isolation is considered outside of the context of pair bonds, which are not reviewed here.

## 7. *Κ* opioids, oxytocin and separation

### (a) Thoughts on mechanisms of separation distress

Results from studies in prairie voles and titi monkeys are interesting in that they suggest an upregulation of OT *over time* following the onset of separation, which is consistent with the human data, and they also suggest that opioid peptides may be involved in the separation distress component of pair bonding. It is likely that during the process of separation and grief, there are dynamic changes in neurobiological processes that vary as a function of time. ***It is our prediction that KOR, as well as central OT release may alter over time with conditions of chronic separation. We predict that central OT release will escape from inhibition and remain elevated while individuals are still under the influence of a social stressor. It is possible that if activated by other mechanisms, KOR may exert influence on central OT release even when CRH remains unaltered or returns to baseline levels*.**

**Figure 1.**
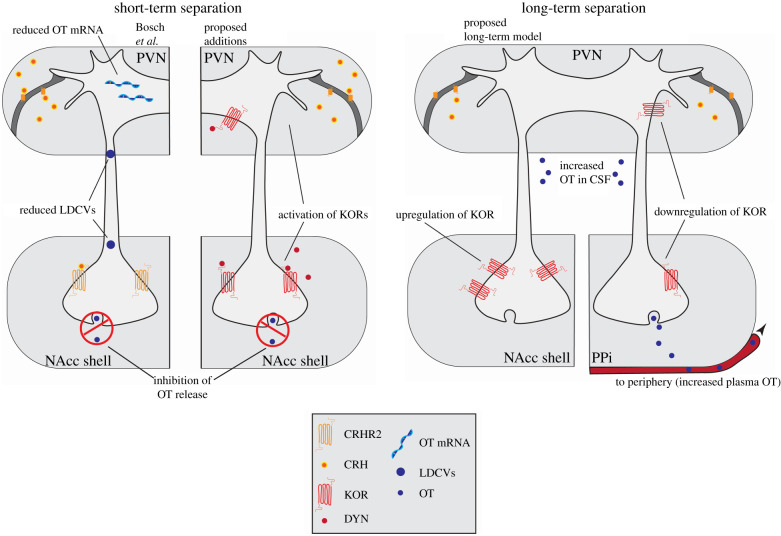
Models for the neurobiology of separation in adult attachment. Here we propose additions to the model proposed by Pohl *et al.* [52]. In short-term separation, KORs may provide an intermediary between CRHR2 activation and reductions in OT that have been found in the NAcc shell. The activation of CRHR2 leads to the release of dynorphin and KOR activation in the PVN and NAcc shell, which in turn has downstream effects on OT that ultimately inhibit OT release in the NAcc shell. We also propose a model for the neurobiology of long-term separation. With long-term separation, we expect downregulation of KORs in the PVN to result in increased release of OT centrally and peripherally. During long-term separation, the continued sense of loss and need to return to a social homeostasis drive increases in CSF and plasma OT, likely due to a decrease in the inhibitory effect of KORs in the PVN. With a reduction of CRH, KORs in the NAcc shell may increase to support the continued aversive state. Abbreviations: KOR, kappa opioid receptor; CRH, corticotropin-releasing hormone; CRHR2, CRH type 2 receptor; DYN, dynorphin; OT, oxytocin; OT mRNA, oxytocin messenger ribonucleic acid; NAcc, nucleus accumbens; LDCVs, large dense-core vesicles; PPi, posterior pituitary.

We suggest the following model (figure 1). (i) During an acute separation, CRHR2 activation leads to dynorphin release and KOR activation in the PVN and NAcc shell. Downstream, KOR effects on OT lead to inhibition of OT release in the NAcc shell. (ii) During long-term separation, the continued sense of loss and need to return to a social homeostasis drives increases in OT (as found in increased CSF OT in titi monkeys, increased plasma OT in humans and increased density of OT immunoreactive cells in the PVN of voles). These increases are likely due to a decrease in the inhibitory effect of KORs in the PVN. If CRH goes down over time, then KOR in the NAcc shell may increase to support the continued aversive state.

The studies above also provide some explanation for why acute partner loss inhibits OT activity in the NAcc, while resulting in elevated CSF levels of OT over the longer term (and in some studies, elevated levels of plasma OT). Chronic social stressors may lead to a decrease in the production of CRH, which could potentially alter both the *κ* opioid and OT systems [108]. Our modified model, based on the work of Pohl *et al*. [52], adds in the role of the *κ* opioid system (figure 1). These models are not mutually exclusive, but rather, our model attempts to add in the role of KORs as a potential mediator of CRH effects on OT while trying to account for adaptations that might occur over longer-term separation, with an eventual, additional goal of better explaining discrepancies in the literature on the neurobiology of separation. An intermediary role for the KORs could explain situations where, for instance, CRH mRNA expression does not differ in paired prairie vole males during separation [44], despite the ability of CRH manipulations to affect passive stress-coping behaviour. In other words, since KORs are downstream of CRHR2, it might be possible for chronic separation stress to exert effects on OT without continued stimulation by CRH, if dynorphin were stimulated by other means.

## 8. Discussion

In this paper, we have proposed an expansion and integration of current models of the neurobiology of pair-bond formation and maintenance. We suggest that the next steps in testing these models would be longitudinal studies of separation and reunion that measure central KORs and changes in OT, and experimental manipulations of each of these systems under conditions of separations of different lengths.

In order to further exploration of this model, it will be necessary to test the direct effects of separation on the *κ* opioid and OT systems, as well as the effects of manipulation of the *κ* opioid and OT systems on the separation response. Current technological restrictions mean that some of these outcome measures are easier to obtain than others, particularly in primates. There is still no commonly available centrally penetrant OT receptor PET ligand [109]. However, there are a number of validated PET ligands for KORs, including one, [C11]GR103545, that has been used in both human [110] and preclinical [111] studies and shown to respond to KOR agonism with a reduction in binding [112]. This reduction in binding is important if it is to be used as a proxy for dynorphin release. Unfortunately, dynorphin itself is extremely difficult to measure *in vivo*. These techniques for measuring dynamic *in vivo* release of dynorphin specifically, and potentially distinguishing between dynorphin fragments, have only recently been made to work in rodents, and only in conjunction with optogenetic stimulation rather than natural ethological stimuli [113]. Karkhanis & Al-Hasani [54], in their 2020 review, give a synopsis of the state of the art in measuring dynorphin release, concluding ‘…we still have a long way to go before we can reliably and consistently measure and distinguish all fragments of *in vivo* dynorphin release during acute and chronic behavioural manipulations…’, although they view the future as very promising. Given these technological limitations, we propose that investigation of the *κ* opioid/OT relationships will, ideally, progress hand-in-hand in rodents and primates, with prairie voles providing a source of more accessible tissue in which to measure changes in gene expression and perform more invasive experimentation, and titi monkeys providing an evolutionary model closer to that of humans.

While extending these studies of separation, two other factors will be important to keep in mind. First, it is obvious from table 1 that many previous pair-separation studies examining these neuropeptides in pair-bonded species have been carried out in males [50]. It will be critical to study both sexes in order to better characterize potential sex differences. For instance, there are sex differences in opioid function in the context of addiction and pain [114]. Some of these differences are dependent on gonadal hormones [115]. In general, morphine does not work as well in women [116]. The mechanism for *κ* opioid analgesia differs by sex, occurring through NMDA receptors in males and melanocortin-1 receptors in females [117]. In prairie voles, KOR activation reduces DA transmission more strongly in males than in females, and a lower dose is required in males [93]. In addition to considering sex differences, it will be important to consider same-sex/gender orientation as well as opposite-sex/gender orientation pair bonds, which are vastly understudied in the pair-bonding literature [81].

It remains important to design studies that allow us to distinguish outcomes specific to the particular experience of partner loss (i.e. grief) from more general experiences of social isolation or loneliness. These distinctions can be made by including treatment groups comprised of non-isolated individuals and/or individuals removed and isolated from a social pairing or group not characterized as a pair bond; for instance, in the prairie vole studies from Bosch *et al*. [44,45], sibling control groups are used. Another research design that allows for this dissociation of the effects of specific loss of the partner versus the general effects of isolation, as employed with titi monkeys [51], uses conditions of reunion with a partner versus encounters with a stranger following separation.

*κ* opioid antagonists have been proposed as treatments for various types of stress-related psychiatric and social conditions, with mixed success [70,118–120]. OT is also often proposed as a treatment for various conditions involving social components [121]. One recent study in rhesus monkeys showed that combining naloxone, an opioid antagonist, with OT had a stronger effect on social attention than the sum of their individual effects [69]. These results lead to the idea that a combination of *κ* opioid antagonist, in concert with OT, could have a stronger effect on grief-related social withdrawal than either treatment alone.

## Data Availability

This article does not contain any additional data.
